# Aquatic Mosses as Adaptable Bio-Filters for Heavy Metal Removal from Contaminated Water

**DOI:** 10.3390/ijms21134769

**Published:** 2020-07-05

**Authors:** Paride Papadia, Fabrizio Barozzi, Danilo Migoni, Makarena Rojas, Francesco P. Fanizzi, Gian-Pietro Di Sansebastiano

**Affiliations:** 1 DISTEBA (Department of Biological and Environmental Sciences and Technologies), University of Salento, Campus ECOTEKNE, 73100 Lecce, Italy; paride.papadia@unisalento.it (P.P.); barozzi.fabrizio@virgilio.it (F.B.); danilo.migoni@unisalento.it (D.M.); makarena.rojas@unisalento.it (M.R.); fp.fanizzi@unisalento.it (F.P.F.); 2 C.I.R.C.M.S.B. Consortium, Villa “La Rocca”-via Celso Ulpiani, 27-70126 Bari, Italy

**Keywords:** moss, phytofiltration, phytoremediation, heavy metals, Hypnales, *Taxiphyllum barbieri*

## Abstract

Heavy metals (HMs) are released into the environment by many human activities and persist in water even after remediation. The efficient filtration of solubilized HMs is extremely difficult. Phytoremediation appears a convenient tool to remove HMs from polluted water, but it is limited by the choice of plants able to adapt to filtration of polluted water in terms of space and physiological needs. Biomasses are often preferred. Aquatic moss biomasses, thanks to gametophyte characteristics, can act as live filtering material. The potential for phytoremediation of Hypnales aquatic mosses has been poorly investigated compared to aquatic macrophytes. Their potential is usually indicated as a tool for bioindication and environmental monitoring more than for pollutant removal. When phytoremediation has been considered, insufficient attention has been paid to the adaptability of biomasses to different needs. In this study the heavy metal uptake of moss *Taxiphyllum barbieri* grown in two different light conditions, was tested with high concentrations of elements such as Pb, Cd, Zn, Cu, As, and Cr. This moss produces dense mats with few culture needs. The experimental design confirmed the capacity of the moss to accumulate HMs accordingly to their physiology and then demonstrated that a significant proportion of HMs was accumulated within a few hours. In addition to the biosorption effect, an evident contribution of the active simplistic mass can be evidenced. These reports of HM accumulation within short time intervals, show how this moss is particularly suitable as an adaptable bio-filter, representing a new opportunity for water eco-sustainable remediation.

## 1. Introduction

Aquatic macrophyte potential for phytoremediation has been investigated in many studies [1], on the contrary the potential of Hypnales aquatic mosses to remove pollutants has been poorly investigated. This is surprising considering that the gametophyte forms dense mats that can be handled and compressed with no significant damage to the organism vitality and thus appear to be a promising filtering biomass.

There are a number of aquatic bryophytes that can accumulate relative high concentrations of contaminants in their bodies [2] and, because of this ability, they have been used as bio-indicators in water. These are non-vascular plants that differ from vascular plants because of their direct uptake pathway via the vegetative thallus. Probably because of this characteristic, aquatic mosses have been shown to accumulate more heavy metals (HM) than vascular plants at contaminated sites. Several studies evidenced the ability of *Leptodictyum riparium* isolated from polluted freshwater to serve as bioindicator, accumulating several heavy metals such as chromium, lead, and zinc [3,4]. *Drepanocladus* spp. [5] and *Warnstorfia fluitans* appeared particularly efficient in the uptake of arsenic [6]. *Funaria hygrometrica* accumulates great quantities of lead [7] but less than 10 species are known for the accumulation of various HMs [8].

Heavy metals are elements having atomic weights between 63.5 and 200.6 but the term also indicates other toxic metals and metalloids [9]. Trace amounts of some of these elements, such as cobalt, copper, iron, manganese, molybdenum, vanadium, strontium, and zinc are needed by living organisms but excess is usually detrimental. Cadmium, chromium, mercury, lead, arsenic, and antimony are non-essential HMs. These can alter significantly biochemical processes in living organisms [10].

HM pollutants derive from several industrial activities, transported by runoff water to contaminate water sources downstream. HM pollution is particularly relevant from metal processing, wastewater from mining, tanneries, pharmaceuticals, pesticides, organic chemicals, plastics, lumber and wood products, etc. [10,11].

In this study the ability to decontaminate water with a high concentration of polluting HMs was tested for the moss *Taxiphyllum barbieri* (obtained from commercial sources). In the short period of time of the experiments, the plants were not morphologically altered by the high concentrations of Pb, Cd, Zn, Cu, As, and Cr.

**Figure 1:**
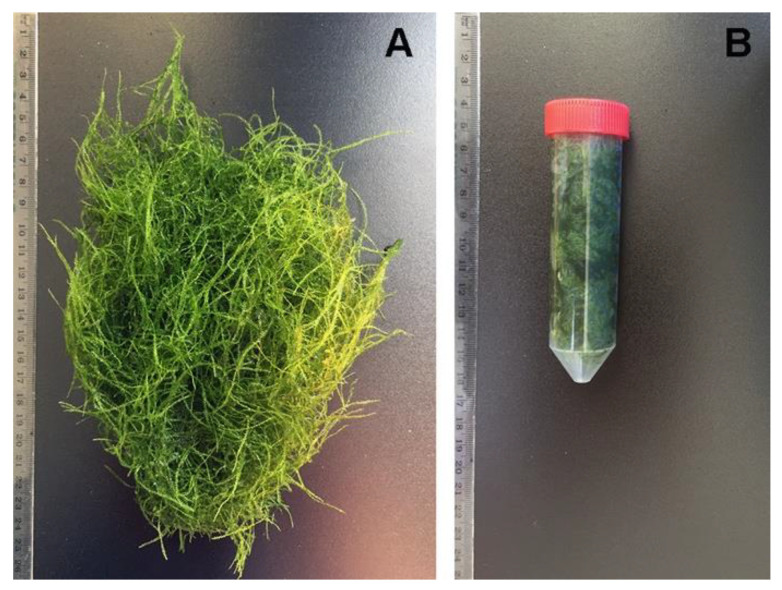
(**A**) A *Taxiphyllum barbieri* 10 g biomass grown in tap water; (**B**) the same biomass can be manipulated and compressed adapting to a smaller volume container with limited damage. Centimeters scale shown on the left of each picture.

Since this plant is largely used as a decorative organism in aquariums, it is known that the thallus grows in very variable light (from 150 µmol/m^2^ s to shaded discontinuous light) and temperature (15–35 °C) conditions. The mechanical properties of the gametophyte mats attracted our attention. They can be removed from water and exposed to air (Figure 1A) for many hours or compressed (Figure 1B) and handled with no significant damage to the organism integrity and vitality (not shown). The moss can be cultivated ex situ and packed to adapt filtering containers of the desired volume and shape (Figure 1B) where it may accumulate significant quantities of HMs within a few hours, representing a new opportunity for water eco-sustainable remediation. In this study, we confirmed the known capacity of mosses to accumulate HMs and then we approached the question of whether moss biomasses can be grown to enhance removal of specific pollutants and be used as filtering material to rapidly reduce water contamination.

## 2. Results

### 2.1. Mosess Grown in Different Conditions Have Different Uptake Capacities

**Figure 2:**
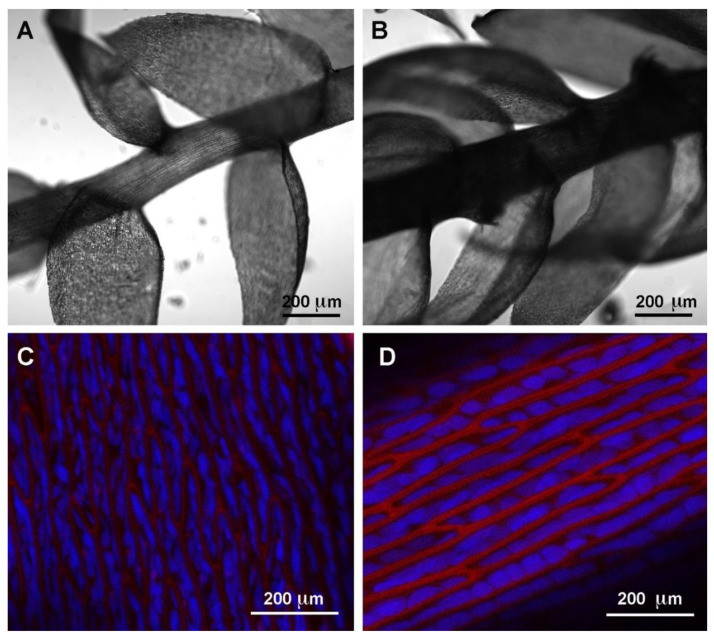
Microscopic images of moss gametophores observed at different magnification. (**A**) Light- green moss gametophore; (**B**) Dark-green moss gametophore; (**C**) Leaflet cells of light-green moss; (**D**) Leaflet cells of dark-green moss. (**A**,**B**) Confocal transmitted-light images; (**C**,**D**) Confocal sections, 3 μm projection showing chlorophyll (in blue) and cell wall autofluorescence (in red). The scale bar length is indicated in each frame.

*Taxiphyllum barbieri* from South-East Asia is a hardy plant which makes few demands on the water quality or light. To test if it can be specifically grown to have different uptake capacities, we tested the performance of mats with two different sets of characteristics. Grown in optimal light conditions *T. barbieri* produces a dark green thallus while under indirect and shaded artificial light the thallus has a light green color. There are two morphological differences in the light-green moss: the gametophore appears elongated, with more spacing of the leaflets (Figure 2A) compared to dark- green moss (Figure 2B) and the chloroplasts in the cells are smaller and the cell wall less autofluorescent (Figure 2C) compared to dark-green moss cells (Figure 2D) suggesting in the latter, a higher ratio between simplastic and apoplastic mass and a different composition in terms of phenolic components of the cell walls.

Samples of 0.5 g drained biomasses with evident color differences were tested in parallel with the same control and contaminated solutions.

**Figure 3:**
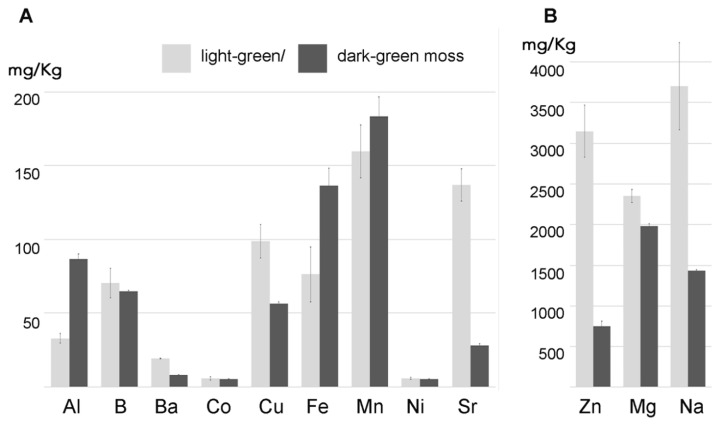
Two moss biomasses, with different colors due to growth in the same medium but different light conditions, accumulating over 24 h several elements with different efficiency (*n* = 3). (**A**) section shows elements represented with less than 200 mg/kg; (**B**) section shows elements represented at higher concentrations. The value in milligrams per kilogram represents the accumulation in control conditions. Error bars represent standard deviation.

In the control solution (1:10 MS) for 12 h, the two moss biomasses absorbed Zn, Mg, and Na with different efficiency; light-green moss was shown to absorb a higher quantity of these elements. In the case of more diluted elements the situation was diversified with dark-green moss adsorbing more Al, Fe, and Mn probably because these elements were required for a higher metabolic activity possibly associated with the higher ratio between the simplastic and apoplastic mass (Figure 3).

### 2.2. Influence of Pectins on HM Absorption

**Figure 4:**
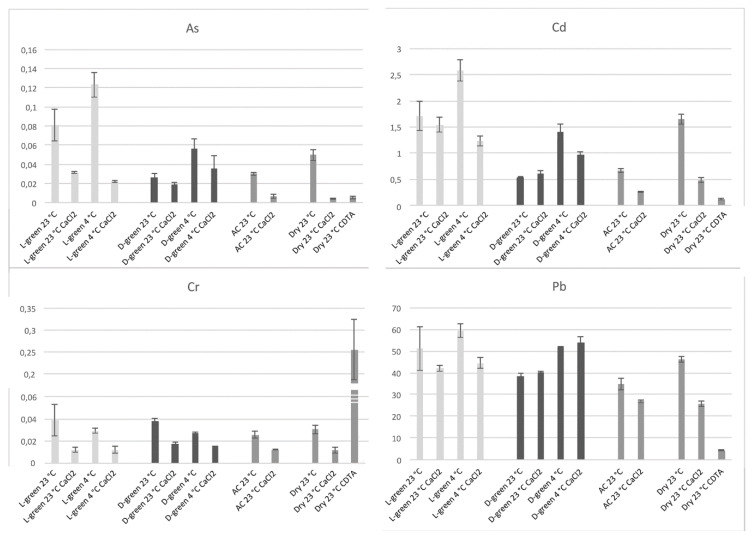
Accumulation of the four elements, As, Cd, Cr, and Pb in moss biomasses treated in different ways. For each element it is possible to see the values, starting from the left, related to light-green moss incubated at 23 °C without washing (L-green 23 °C), after washing with CaCl_2_ (L-green 23 °C CaCl_2_), incubated at 4 °C without washing (L-green 4 °C), after washing with CaCl_2_ (L-green 4 °C CaCl_2_), dark-green moss incubated at 23 °C without washing (D-green 23 °C), after washing with CaCl_2_ (D-green 23 °C CaCl_2_), incubated at 4 °C without washing (D-green 4 °C), after washing with CaCl_2_ (D-green 4 °C CaCl_2_), autoclaved light-green moss incubated at 23 °C without washing (AC 23 °C), after washing with CaCl_2_ (AC 23 °C CaCl_2_), dry light-green moss incubated at 23 °C without washing (Dry 23 °C), after washing with CaCl_2_ (Dry 23 °C CaCl_2_), after CDTA extraction (Dry 23 °C CDTA). Data are expressed as mg/kg of dry weight. Error bars represent standard deviation (*n* > 4).

We analyzed the amount of As, Cd, Cr, and Pb accumulated in moss biomass under different temperature conditions (23 °C and 4 °C) and after different washes which were able to remove elements associated with polysaccharides. In particular, in Figure 4 it is possible to observe the accumulation of the four elements in light-green or dark-green moss at 23 or 4 °C and the effect of washing with CaCl_2_. It is then possible to observe the accumulation in an autoclaved biomass, deprived of an active protoplast, and the accumulation in a dry biomass, after washing with CaCl_2_ and after pectin removal through CDTA extraction.

The great importance of the interaction with cell wall polysaccharides was confirmed but it was also possible to note, in addition to small differences between light and dark-green mosses, that Cr and Pb are accumulated by different mechanisms. Cr is evidently bound to a cellular fraction different from pectins since CDTA extraction dramatically increases Cr concentration in the residual sample; Pb accumulation is probably influenced by uncharacterized cellular processes because washing with CaCl_2_ did not reduce the concentration only in the case of dark-green moss.

**Figure 5:**
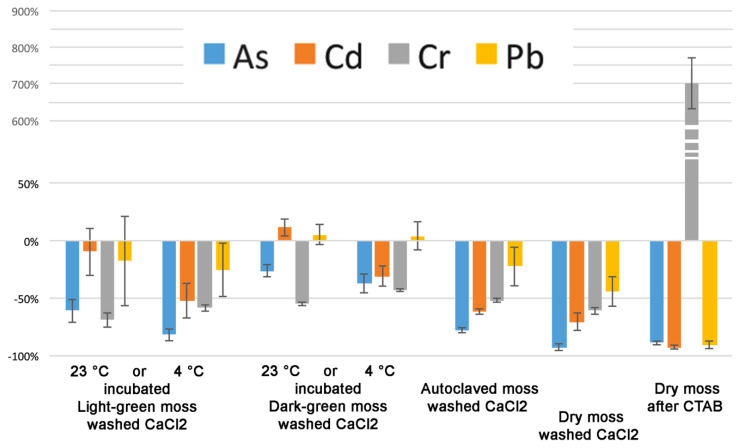
As, Cd, Cr, and Pb percentage change due to washing treatment after accumulation at different temperatures. The analysis is based on Figure 4 data. Error bars represent standard deviation.

In general, the effect of washing is different between light- and dark-green mosses. In Figure 5 this effect, expressed as a percentage change, is better represented. In particular, Cd and Pb accumulated at 23 °C were not washed away by CaCl_2_ so easily as that accumulated at 4 °C and dark- green moss appeared to retain these elements better.

### 2.3. Moss Grown in Low Light Has Better Performance in Absorbing Pb

Zn and Cu, are important elements that may also be representative of relevant contaminants in polluted water. They are translocated by land plants with diverse mechanisms [12]. We considered it relevant to analyze their influence on the accumulation of more dangerous pollutants when comparing dark- and light-green moss performance. In this way we also investigated the effect on physiological processes normally involving Cu and Zn. Mosses were treated with As (20 µM) alone, Zn (200 µM) alone or the two chemicals combined to test interactions as well as with Cu (20 µM) alone, Cd (50 µM) alone, Pb (500 µM) alone or the combination of Cu with Cd or Pb.

HMs accumulation in a 12 h period was similar in both kinds of biomass with the only evident difference being in the accumulation of Pb which was much lower in dark-green biomass. When moss was treated with Pb and Cu, the Pb accumulation in dark-green moss increased, reaching the performance of light-green moss. As and Cd accumulation was anyhow much lower than Pb (Figure 4).

### 2.4. Diversified Kinetics of HM Subtraction from Contaminated Solution

**Figure 6:**
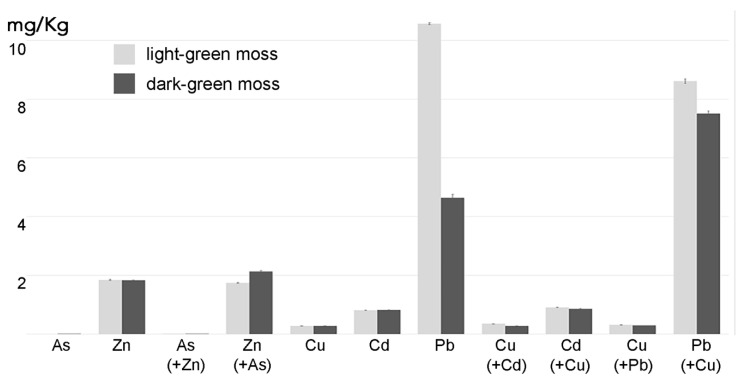
Concentration of As, Cd and Pb, alone or combined with Zn or Cu, in two moss biomasses with different colors (*n* = 3) after 24 h incubation in diluted MS supplemented with the indicated elements. The data expressed as grams per liter represent the amount of element detected above the background concentration found in control mosses grown in parallel in diluted MS. The values expressed by the histogram columns refer to the element indicated at the column base, the element in brackets indicates the co-treatment and its value is expressed by the adjacent histogram columns. Error bars represent standard deviation.

**Figure 7:**
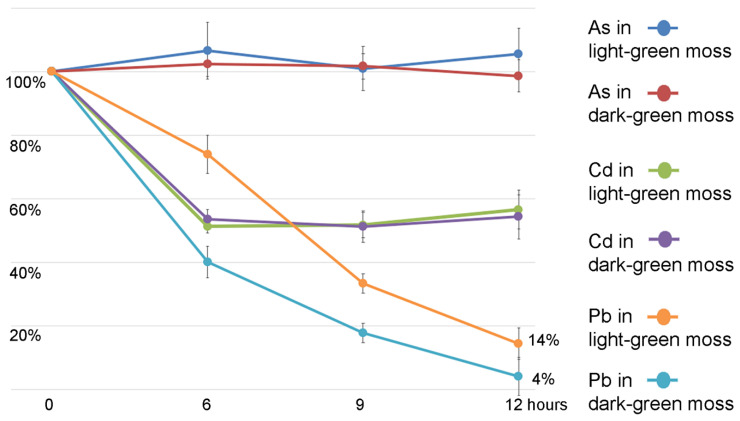
Concentration reduction of the HMs in the liquid samples collected from the experiment presented in Figure 6 at different time intervals (6, 9, 12 h). Data are expressed as percentage of the HM treatment at time 0. The two kinds of light- or dark-green biomass and the element measured are distinguished by colors. Error bars indicate standard deviation (*n* = 3). Variation in As concentration is not significant even compared to control conditions. Cd reduction has no statistical differences when comparing the two kinds of moss. Pb concentration change is differentiated in different moss kinds.

To test HM absorbance kinetics, liquid samples from the experiment presented in Figure 6, were collected at time 0, 6, 9, and 12 h. The HM concentration reduction, expressed as percentage of the HM treatment, was monitored. As^3+^ (NaAsO_2_ 20 µM) concentration was not significantly reduced (Figure 5). Cd^2+^ (Cd(NO_3_)_2_ 50 µM) concentration was reduced only within the first 6 h reaching some kind of saturation, while Pb^2+^ (Pb(NO_3_)_2_ 500 µM) concentration continued to be reduced in time with differences between light- and dark-moss, in line with the absorbance observed in the plant mass at the end of the 12 h period (Figure 7).

### 2.5. Moss Short Term Cd and Cr Filtering Ability

The ability of samples of 0.5 g of drained mosses to filter Cd^2+^ (Cd(NO_3_)_2_ 0.15 mg/L) and Cr^6+^ (K_2_Cr_2_O_7_ 1 mg/L) contaminated tap water was compared with the filtering effect of 0.5 g synthetic nylon filtering material for aquariums (FLO, Askoll, Germany). Being nearly inert, such filters could be considered a background control. To test the performance of moss cultivated for different periods of time, three replicas of the experiments were performed using moss cultivated in independent tanks for different periods of time, in particular, 3, 6, and 9 months, without evident differences in the performance, showing that the accumulation potential is not related to the mat age.

**Figure 8:**
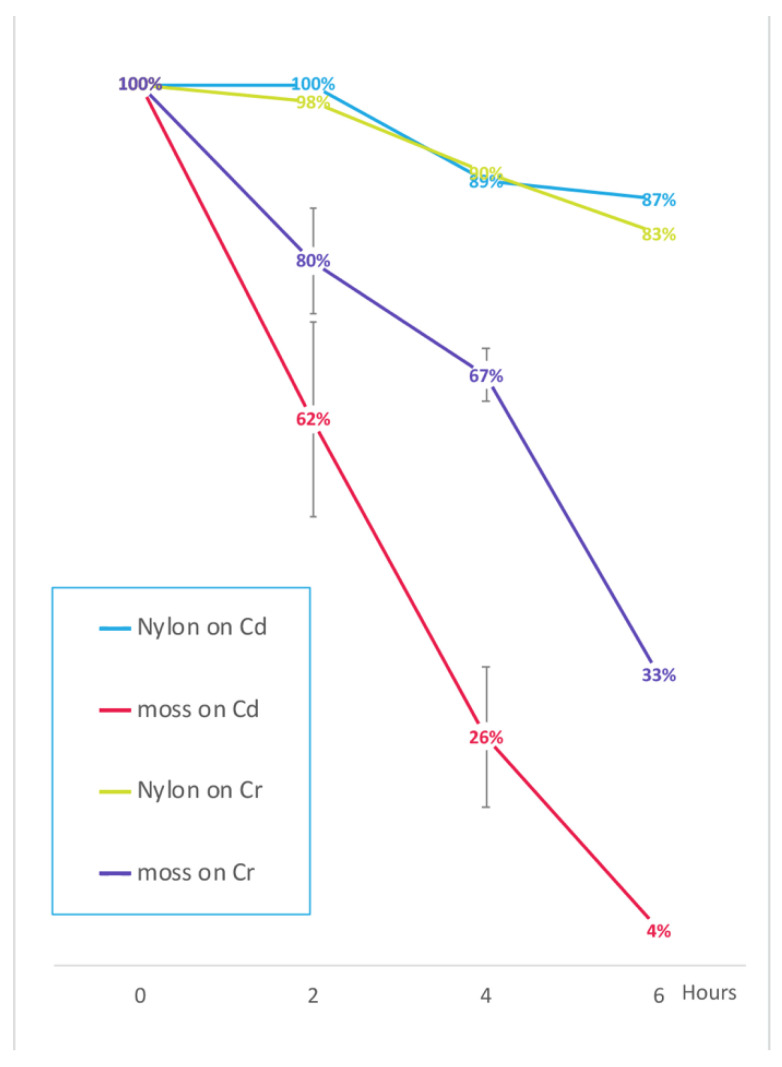
Concentration reduction of the HMs in contaminated water “filtered” by light-green moss compared to nylon filtering material. Data are expressed as percentage of the HM treatment at time 0. Cd contamination was obtained with 0.15 mg/L Cd(NO_3_)_2_ and Cr contamination was obtained with 1 mg/L K_2_Cr_2_O_7_. Error bars indicate standard deviation (n = 3). For the 3 replicas independent moss cultures and different ages (3–6–9 months) were used. The reduction observed after nylon filtration represents a background effect possibly independent of the filtration procedure.

Moss ability to uptake Cd reached its maximum after 6 h (Figure 8) as already evidenced by previous analysis. It was possible to calculate the uptake of about 4.3 g cadmium per kg dry weight (DW obtained after 3 days at 50 °C) of moss within six hours.

In the same period moss reduced Cr concentration to 33% of the initial concentration (Figure 8). It was possible to calculate the uptake of about 19.4 g chromium per kg of DW moss within six hours.

## 3. Discussion

Heavy metals are released by many industrial activities and may be residual in water after different forms of remediation. Filtering of solubilized HMs is extremely expensive. Membrane filtration technology is preferred for its simplicity, energy efficiency, and manufacturing scalability [13]. However highly concentrated minerals such as calcium sulphate and carbonate may deposit on the membrane causing so called “scaling” [14], leading to increased flow resistance, requirement for frequent cleaning, and replacement of membranes. To reduce scaling, more expensive membrane modifications are needed [15].

Because of the relative high cost of membrane filtration, phytoremediation appears to be an eco- friendly and economic alternative to remove HMs and metalloids from polluted water. However, the success of phytoremediation depends upon the identification of high biomass, metal tolerant plants.

Another problem is the adaptability of plants to existing technologies and approaches that usually implies a relatively fast flux of polluted water. The concept of pollutant sequestration in the growing biomass may sometime not be applicable when the growing rate is not in line with the wastewater flux.

Aquatic mosses cultivated ex-situ can be packed to adapt filtering containers of the desired volume and shape. Some of them have extremely coherent and resistant thalli so that they do not release debris, representing also a valid mechanical filter for particles. At the same time, they can be a natural support for the growth of bacteria usually part of bio-filtration, replacing less eco- friendly artificial materials [11].

To the best of our knowledge, this is the first assessment of HM phytofiltration capacity of live aquatic moss species among the most versatile and resistant, *Taxiphyllum barbieri.* It has no problematic requirements to grow and, as other similarly resistant species such as *Vesicularia dubyana*, can represent an organism adaptable to several filtering needs of modern technologies.

We tested the ability of two independently cultured mats of *Taxiphyllum barbieri* to capture some of the most dangerous HMs such as As, Cd, Pb, and Cr. We used two biomasses grown under different light conditions, showing different morphological characteristics, with the intent to answer the question of whether the same organism could be grown to better respond to different remediation needs. The morphological characteristics we monitored are shown in Figure 2. They behave differently, accumulating various elements in a differentiated way. We show this in Figure 3, by analyzing the accumulation of elements normally present in the culture medium used during the experiments.

Since dry moss biomass was previously shown to be a valid biosorbent for Cd [16], we evaluated if differently grown mosses accumulated elements because of interaction with pectins or because of other processes. We used washing with CaCl_2_ to extract weakly bound elements or CDTA to remove pectins completely.

In live cells, metal uptake is considerably affected by variation in temperature due to the dependence on metabolism, even, biosorption, which is independent of the cell metabolism mechanism, is also affected because it involves sorption onto the cell surface based on physical and chemical interactions between metal and functional groups of the cell wall [17]. As well evidenced in *Zooglea ramigera*, the sorption of Cu(II) and Ni(II) ions is increased with decreasing temperature but the sorption of Pb(II), Cr(VI) and Fe(III) ions increased with increasing temperature [18]. Bond rupture, caused by increasing temperature, enhances the number of active sites involved in metal sorption or the higher affinity of sites to metals has been ascribed to increased biosorption [19]. Similarly, Kayalvizhi et al. (2014) reported that the biosorption capacity of an algal biomass, could be increased with an increase of temperature. The pores in the biomass become enlarged favoring the sorption process and diffusion of Cr(VI) ions [20]. To explore the influence of this parameter, two different temperatures were used to incubate live moss: 23 and 4 °C.

Our results, reported in Figure 4 show that despite the fact that most of the accumulation can be related to pectins, the differences between light- and dark-green moss are too important to depend entirely on the cell wall. Cr is evidently bound to a cellular fraction that is different from pectins. The CDTA extraction increased Cr concentration in the residual sample. Lead accumulation appeared to be related to uncharacterized cellular processes, different from ionic binding, but more efficient in dark-green moss. In general, as better evidenced by Figure 5, the effect of washing treatments was different between light- and dark-green moss.

Reassured of the physiological difference of these differently cultivated biomasses we also analyzed the effect of zinc and copper co-accumulation on the accumulation of some more toxic pollutants. In fact, regardless of their toxicity or physiological role, the variations in uptake of some elements can influence the uptake and accumulation of other elements with synergistic or antagonistic effects.

A stimulating effect of Fe deficiency was observed in *N. caerulescens* on Cd uptake and it was found to be connected with Fe-regulated transporter-like protein genes [21]. Among the nutrient elements, Ca is related to Cd in *B. juncea* and *S. alfredii* [22,23]. Cd concentration in *N. caerulescens* plants correlated also with Zn, Fe, and Cu concentrations [24] and Zn uptake was reduced by Cu excess in soil [25].

If aquatic mosses are to be used for water filtration and remediation, their behavior in the presence of multiple pollutants should be better investigated. In our study we can visualize that higher Cu concentration may positively correlate with increased Pb uptake by moss grown in optimal light. In general, our experimental setting did not allow to distinguish if element accumulation was due to uptake or adsorption but when differences correlate with treatments or gametophyte morphology, as shown in Figure 6, a physiologic active contribution can be hypothesized and we may suggest an increased uptake under specific conditions.

The most interesting observation remains the different behavior of the two mats cultivated in different conditions. These developed diversified morphological aspects suggesting possible physiologic differences that need to be investigated further.

Several moss species have been found particularly efficient for HM accumulation [7,8] but in our work we suggest that each moss species could be cultivated differently so as to be adapted to the accumulation of different pollutants. Differences may be due to different accumulation capacity in the cell wall or intracellular compartments [26] depending on physiologic adaptations.

In the experiments reported in Figure 7 and Figure 8, the removal of HMs from the liquid medium was reported. As, Cd, and Pb removal was tested in a period as short as 6 h, up to 12 h. Arsenic (As(III)) was not removed from the liquid medium. Further experiments are needed to elucidate its behavior with both arsenite and arsenate uptake. The analysis of Cd and Pb removal in different periods of time, reported in Figure 7, showed that even if the Pb total accumulation capacity in proportion to biomass is much higher than for Cd, the initial efficiency speed is similar.

We then proposed an experiment where Cd removal from liquid medium was analyzed at shorter time intervals (2 h), and compared to Cr(VI), it is another extremely important pollutant [26]. Using a ten times larger biomass the HM removal appeared not to be influenced by saturation limitations and also showed how fast removal can be. A rapid wastewater phytofiltration appears to be an appealing possibility.

Phytofiltration capacity of an aquatic moss for application in polluted water remediation as a bio-filter, has only started to be investigated moving research to further its value as bioindicator [6]. It was shown that the aquatic moss, *Warnstorfia fluitans* took up As from water accumulating up to 4.5 mg of As per g of dry weight (DW) without influencing its biomass [6]. The moss accumulated high levels of As similar to those found in As-hyperaccumulating fern, *Pteris vittata*, which accumulated 3.5 mg As per g DW in its fronds without any sign of toxic effects [6,27].

A study found that another submerged aquatic plant species, *Vallinseria natans* (Lour.) Hara, accumulated nearly 2 mg As per g DW when it was exposed to 5 mg/L arsenate for 7 days [23]. Within a similar time period *W. fluitans* had a higher As accumulation than *V. natans*. It might be possible to use the term As “hyperaccumulator” here, as it refers to plants accumulating over 1 mg per g DW in their aboveground parts [28,29]. In aquatic moss, however, there is direct uptake via the thallus and HMs do not reach the green parts via root transport. The direct uptake by the whole plant body in submerged plants probably explains the higher concentrations found in them than in terrestrial and emergent plants [30].

We need to consider that the studied moss plants here originated from a commercial source and were not optimized for phytoremediation. Plant clones could be further improved genetically through controlled growth under selective pressure to stimulate the up-regulation of resistance mechanisms [31].

Sandhi and co-authors (2018) reported that the As concentration in *W. fluitans* increased with increasing As concentration [6], as found also in other studied species [32]. The relationship between the internal and external concentrations was linear, at least up to the 100 mM arsenate (As(V)) treatment: in our case we used arsenite (As(III)) and this is the possible reason why the uptake was irrelevant. Indeed it is normal to have lower uptake of As(III) than As(V) in emergent and terrestrial plants [30]. Anyhow the opposite could also be observed in submerged plants, for example in *Spartina* spp. [33] As uptake was higher from arsenite than from arsenate-bearing solution. At least we evidenced here the tolerance of moss to As treatment. Further studies are certainly required to establish the potential As(V) uptake.

To summarize, this work demonstrated that *T. barbieri* would be a suitable candidate for the phytofiltration of Pb (>100 g/kg DW in 6 h), Cd (about 4.3 g/kg DW in 6 h) and Cr (about 19.4 g/kg DW in 6 h) from contaminated water since it has high and rapid accumulation capacity for these HMs. A comprehensive comparison with other technological methods should include a long term environmental impact but this is too difficult to obtain. Nonetheless the comparison with an equivalent mass of synthetic nylon, provided here an interesting indication.

The high removal rate from the water of some HMs has to be coupled with the easy cultivation and the strong thallus that can work as support for nitrifying bacteria. This makes *T. barbieri* a very good candidate for phytoremediation/phytofiltration of water polluted by specific human activities or for complete water remediation downstream from other purification processes.

## 4. Materials and Methods

### 4.1. Plant Material

The aquatic moss *Taxiphyllum barbieri* was acquired from commercial source (Tropica Aquarium Plants; Mejlbyvej 200 8250 Egå, Denmark) and grown in tap water with no supplements and moderate aeration (to prove economical of its cultivation). The temperature of the growing chamber was 22 °C and the light intensity during a 16 h light period was variable: optimal, 150 µmol/m^2^ s, to grow dark-green gametophyte, or shaded (the moss tank was placed in the shade and received indirect light only) to grow light-green gametophyte.

The growth of the moss was slow but continuous with a mass increase of about 10% per month without nutrients added. Live moss mats were manually collected. In the majority of the experiments the biomasses derive from a single moss mat of each morphology but in the experiment reported in Figure 6 gametophytes cultivated for different periods of time (3–6–9 months) were used to take into consideration the variability due to mat age.

The moss samples were washed with distilled water to remove plant material debris and distributed under submerged conditions in separated 50 mL Falcon tubes for phytoextraction experiments.

The moss used in the experiments was drained but not dried. Gametophyte was gently pressed between adsorbing paper till the paper remained dry. We tested that drained moss remains vital with unchanged characteristics for at least 6 h. Five grams of drained moss corresponds to about 0.5 g dry weight (DW). In particular 5 g drained dark-green moss corresponded to 0.48 ± 0.04 DW, 5 g drained light-green moss corresponded to 0.5 ± 0.08 DW.

### 4.2. Microscopy

Gametophyte was imaged at high magnification by confocal laser scanning microscope LSM 710 Zeiss (ZEN Software, Oberkochen, Germany). Excitation wavelengths for autofluorescence were 488 and 543 nm. Cell wall fluorescence emission was collected in the range of 560–615 nm. Chlorophyll fluorescence emission was collected over 650 nm.

### 4.3. Metals Accumulation Treatment

Moss fragments of 0.5 g were moved in 50 mL Falcon tubes containing 1:10 diluted liquid MS (0.44 g/L; Murashige and Skoog medium including Gamborg B5 Vitamins; Duchefa, Haarlem, The Netherlands) as control (shown in Figure 3) or supplemented with HMs and metalloid.

In the experiment represented in Figure 4, test-tubes were supplemented with a mixture of As^3+^ (NaAsO_2_) 20 µM, Pb^2+^ (Pb(NO_3_)_2_) 500 µM, Cd^2+^ (Cd(NO_3_)_2_) 50 µM, Cr^6+^ (K_2_Cr_2_O_7_) 10 µM, in combination. As a control, autoclaved and dry moss were used under the same conditions previously described. The tubes were placed at 23 °C or on ice (4 °C) depending on the treatment over 24 h. Then, some samples were washed with ice cold 5 mM CaCl_2_ to evidence the low affinity binding of the heavy metals in the cell wall.

Solutions of As^3+^ (NaAsO_2_ 20 µM), Zn^2+^ (ZnSO_4_ 200 µM), Cu^2+^ (CuSO_4_ 20 µM) Cd^2+^ (Cd(NO_3_)_2_ 50 µM) Pb^2+^ (Pb(NO_3_)_2_ 500 µM) were administered alone or in combination (final liquid volume 40 mL). As was combined with Zn, Cd and Pb were combined with Cu. Figures indicate clearly the element represented in the graphic while the second element, supplemented but not shown in the graphic, is indicated in brackets.

When treatments are applied the concentration refers to the concentration above the background of an untreated sample. Background was usually very low except in the case of Zn. To explain the presence of elements not included in the diluted MS, please consider that the moss was grown in non-sterile tap-water possibly contaminated by the laboratory practice. This makes the establishment of background values very important for the following analysis.

Three samples of 1 mL of liquid MS were collected from each treated moss at 0 h, 6 h, 9 h, and 12 h intervals. Three additional samples of 1 mL liquid MS were collected at time 0 h as control condition. The moss in control condition was immediately washed two times with distilled water and air dried for 1 week while the mosses in the treated condition were collected and dried in the same way at the end of treatment.

### 4.4. Cyclohexane Diamine Tetraacetic Acid (CDTA) Extraction of Plant Cell Wall Pectin

The extraction of pectin was carried out following the protocol described by Bethke and Glazebrook (2014) [34], with some modifications. Frozen moss at −40 °C for 4 days was triturated in a mortar on ice, 10 mL of pre-heated CDTA extraction buffer was added in a Falcon 50 mL tube with the triturated moss, after incubation, the sample was centrifuged at 4500 rpm for 15 min, the supernatant was removed and the pellet was dried at 37 °C for 4 days.

### 4.5. Filtration Simulation

Biomasses of 5.0 g of live moss were moved in 50 mL Falcon tubes containing 40 mL of water contaminated respectively with 0.15 mg/L Cd(NO_3_)_2_ or 1 mg/L K_2_Cr_2_O_7_. Each treatment was replicated 3 times. As a control a 2.0 g mass of synthetic nylon filtering material for aquariums (FLO, Askoll, Germany) was used. Nylon was selected as negative control because it could represent a mechanical filter only. Fluctuation in HM concentration may be considered as a sort of background. At the beginning of each experiment replica (time 0), 1 mL of liquid was collected from each Falcon tubes, while after 2 h, 4 h, 6 h 3 samples of 1 mL of liquid were collected. The percentage value reported in Figure 8 was calculated based on the sample value at time 0.

### 4.6. Mineralization and Metal Accumulation Analysis

The dried mosses were mineralized in a microwave in the presence of concentrated ICP-grade HNO_3_ and H_2_O_2,_ respectively 6 mL and 4 mL. Then 900 µL of each liquid sample collected, was acidified with 500 µl of concentrated ICP-grade HNO_3_ and diluted to a final volume of 5 mL with HNO_3_ (2% v/v). All mineralized samples were analyzed using ICP/AES (iCAP 6000, Thermo Scientific, Waltham, MA, USA). The spectrometer was previously calibrated for quantitative analysis with five standard solutions (Ultra Scientific Analytical Solution, Bologna, Italy) containing known concentrations of the elements: 0.001, 0.01, 0.1, 0.5, and 1.0 mg/L. The calibration lines showed correlation coefficients (r) greater than 0.99 for all the measured elements. The results were expressed as the average of three different measurements, and the element concentrations were expressed as ppm (mg/kg of sample weight) in solid samples, and as mg/L in liquid samples. All experiments were repeated independently 3 times. The *p* values for statistical significance (*p* < 0.05) were obtained using two samples *t*-test function integrated in the OriginPro 2017 suite. The values differences are often statistically significant but, dealing with a small number of replicates and comparing only two independent cultures of moss, we deemed it was better to discuss only very evident differences from the graphs.
